# Diagnostic value of iris biopsies for the assessment of iris pigment changes and diffuse iris melanoma (DIM) in cats: A descriptive histopathological study

**DOI:** 10.1177/03009858251403170

**Published:** 2025-12-31

**Authors:** Sabine Beetz, Robert Klopfleisch, Carolina Naranjo, Emma Scurrell, Judith Bushe, Annika Lehmbecker, Gerhard Loesenbeck, Sophie E. Merz, Hannah Pischon, Ingo Spitzbarth, Birgit Müller, Katharina Thieme, Sixta Wellen, J. Corinna Eule

**Affiliations:** 1 Freie Universität Berlin, Berlin, Germany; 2 IDEXX Laboratories, Barcelona, Spain; 3 Cytopath Ltd., Ledbury, UK; 4 Antech Tierpathologie München, Munich, Germany; 5 IDEXX Laboratories, Kornwestheim, Germany; 6 Laboklin GmbH & Co. KG, Bad Kissingen, Germany; 7 Tieraugenzentrum am Neckar, Dossenheim, Germany; 8 Tierärztliches Augenzentrum Frankfurt-Kalbach, Frankfurt, Germany

**Keywords:** feline, hyperpigmentation, iris biopsy, iris melanoma, iris melanosis, ophthalmology

## Abstract

Feline diffuse iris melanoma (FDIM) is a common ocular neoplasm, typically affecting older cats, characterized by progressive hyperpigmentation and potential malignancy. This study evaluated the diagnostic value of iris biopsies in distinguishing FDIM from benign iris melanosis. Seventeen cases with suspected FDIM were analyzed through ex vivo iris biopsies and corresponding enucleated globes. Histological evaluation by 8 pathologists revealed an average concordance of 81.6% between biopsy and globe diagnoses. The reliability of biopsy samples was influenced by tissue quality, with 12/136 slides deemed non-diagnostic due to artifacts or insufficient tissue. While biopsy retrieval offers a less invasive alternative to enucleation, its effectiveness is limited by technical factors, including tissue handling and sampling techniques. The results suggest that iris biopsies can be valuable for diagnosing advanced FDIM but require skilled execution. Further studies should explore early-stage FDIM and melanosis to better assess the diagnostic potential of biopsies across various disease stages.

The most common primary uveal neoplasm observed in cats is diffuse iris melanoma (DIM).^4,6,12^ This condition originates from melanocytes lining the anterior iris stroma^7^ and is one of the 2 most common manifestations of melanocytic tumors in cats, along with the cutaneous form.^3,19^ Typically affecting cats over the age of 9 years,^12,15,20^ the age of affected animals spans from 2.8 to 23.1 years.^20^ There is no recognizable predisposition based on sex or breed.^12,20^

Feline diffuse iris melanoma (FDIM) often arises from a benign stage, such as melanosis or benign hyperpigmentation.^10^ During this benign stage, focal or multifocal hyperpigmented areas on the iris surface, known as iris nevi,^9^ are observed. These areas may vary in number and size and remain unchanged over an extended period. Iris melanosis is typically non-elevated, displaying a color spectrum from light to dark brown, and does not impact pupil mobility or intraocular pressure (IOP).^2^ Histologically, melanocytes in this stage are exclusively located on the anterior surface of the iris.^7^ These nevi can progress to iris melanomas through the proliferation of neoplastic melanocytes.^13^ The growth rate or progression to iris melanoma varies significantly, with some nevi exhibiting slow or no growth over years, while others change quickly.^6^ Predicting the progression course is challenging,^5^ although nevi in young cats tend to undergo changes more rapidly. Clinically, changes are typically multifocal or diffuse, presenting a color spectrum ranging from amber to light and dark brown and black.^10,13^ The definitive assessment of the transition from melanosis to an early form of FDIM can only be made through histological examination, marked by the invasion of dysplastic melanocytes into the iris stroma,^5,20^ in which pigment cells gradually extend into the ciliary cleft, the trabecular meshwork, and the ciliary body, and in some cases, penetrate the sclera and/or choroid. Clinically, this can result in iris thickening, impaired pupil motility, and the development of secondary glaucoma.

Fuchs et al^9^ introduced a clinical classification system, termed the “preoperative pigmentation severity score” (PPSS), to characterize the extent of pathological pigmentation. Differentiating between iris nevi and the 5 severity grades of FDIM is achieved through inspection, assessment of pupil movement and isocoria, slit-lamp examination, and tonometry.

Nonetheless, there remains a diagnostic challenge in reliably distinguishing clinically between iris melanoma and melanosis, given the subjective nature of the described grades 0–3 by Fuchs et al.^9^ Since early-stage iris melanoma cannot be reliably distinguished from melanosis by clinical examination alone, diagnostic approaches for detecting advanced melanoma are used before discussing treatment options with the cat owner (eg, laser treatment, enucleation). These include aqueous humor analysis and iris biopsies. While aqueous humor collection is routine in human medicine for patients suspected of metastasis due to iris melanoma,^8^ it has infrequently yielded diagnostic results in previous veterinary studies.^14,16,21^

The study by Featherstone et al^7^ aimed to demonstrate the benefits of iris biopsies in cats, specifically in distinguishing between melanosis and early FDIM. In that study, 6 biopsies were examined. Of these, iris melanosis was detected in 3 cats. Only 2 cats showed FDIM and 1 sample was not diagnostic. Building on the assertion by Featherstone et al^7^ that iris biopsies can be performed safely, this study aimed to assess the reliability of these biopsies. It also sought to evaluate the agreement between the histological diagnosis of iris biopsies and the corresponding enucleated globe to determine whether both samples yield the same diagnosis.

## Material and Methods

This is a prospective ex vivo study.

### Case Selection

The following inclusion criteria were established: clinical suspicion of FDIM, documentation of clinical and ophthalmological findings, enucleation of the affected globe, and extraction of iris biopsies. Data such as breed, age, sex, observation period, number of hyperpigmented areas, distribution patterns, size, color, iris thickening, restriction of pupillary movement, gonioscopy findings, IOP, and additional screening tests like blood tests, abdominal ultrasound, and chest X-rays were collected. Exclusion criteria were defined as cases that had missing patient information, poor histological tissue quality, and/or unclear histopathological diagnoses.

### Specimens

**Figure 1.**
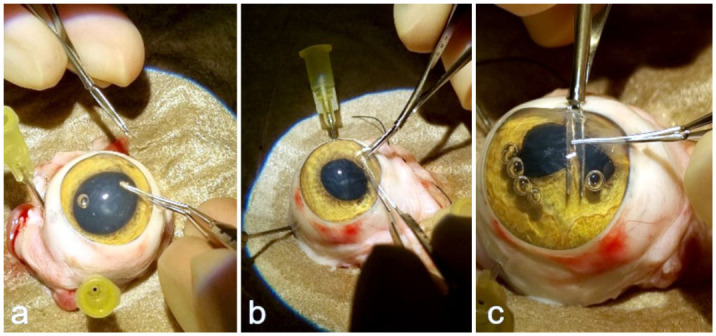
Iris biopsy from an enucleated globe of a cat without evidence of feline diffuse iris melanoma. After 2 clear cornea stab incisions, the anterior chamber was filled with viscoelastic (a), the iris was grasped using a delicate forceps (eg, Utrata) and gently lifted (b), and excised using iris scissors (c).

After enucleation, 2 to 4 iris biopsies were taken from each globe (Fig. 1). All biopsies and globes were fixed in 7% neutral-buffered formalin, routinely processed into 3 to 4 tissue blocks, and embedded in paraffin. This allowed for the generation of 1 to 3 µm thick sections. The slides were then stained with hematoxylin and eosin.

A board-certified pathologist (Diplomate of the American College of Veterinary Pathologists) from the Institute of Veterinary Pathology at the Freie Universität Berlin examined all tissue blocks of each globe and biopsy histologically (2–4 slides per eye) and selected a representative tissue block of each tissue containing relevant lesions. From these blocks and the corresponding biopsies, 2 slides each were prepared, with one being directly stained with hematoxylin and eosin and the other undergoing bleaching before hematoxylin and eosin staining. The bleached slides were included to facilitate the identification of mitotic figures and the assessment of the mitotic index, which can be obscured in heavily pigmented tissues.

The total sample pool comprised 21 cases. Of these, 17 fulfilled the inclusion criteria and were subsequently subjected to final evaluation, comprising 2 cases of melanosis, 7 of early-stage FDIM, and 8 of mid-/advanced-stage FDIM. These diagnoses were based on the initial assessment of the entire globes by the routine pathology diagnostic service. All slides were digitalized using a linear scanner (Aperio AT2, Leica) to create whole-slide images. They were scanned at a magnification of 400× (image resolution 0.25 µm/pixel).

### Participants and histopathologic evaluation

Digital slides were provided to 8 board-certified pathologists (Diplomates of the American and/or European Colleges of Veterinary Pathologists). Six of the 8 pathologists had no focus in ocular pathology; 2 pathologists were specialized in ocular pathology. The samples were blinded to the examiners, so it was not clear which iris biopsy belonged to which globe. The pathologist who performed the initial histological examination of the globes and selected the slides did not take part in the subsequent blinded evaluation of the biopsy samples. Pathologists received a multiple-choice questionnaire for each sample, covering degree of invasion, arrangement of tumor cells, pigmentation, mitotic count, cellular pleomorphism, nuclear shape, and final diagnosis. Different questionnaires were used for biopsies and globes. Biopsy questionnaires sought information on sample analyzability and the presence of suspicious areas for FDIM. Response options for the final diagnosis varied, with the globe questionnaire offering more detailed choices (no lesion, iris melanosis/benign hyperpigmentation, early FDIM, mid-stage FDIM, advanced FDIM) compared to the biopsy questionnaire (no lesion, melanosis/benign hyperpigmentation, FDIM). Pathologists had the option to abstain or provide comments at each query point. The histological grading of the samples was based on the classification by Dubielzig,^4^ who adapted the staging system originally proposed by Kalishman et al^12^ for FDIM by adding iris melanosis as a separate, non-neoplastic category. This system categorizes uveal melanocytic tumors into 4 stages: “iris melanosis,” where pigmentation is limited to the iris surface; “early FDIM,” characterized by neoplastic cell invasion into the iris stroma and anterior chamber; “moderate FDIM” (or “mid-stage”), where neoplastic cells extend into the ciliary body; and “advanced FDIM,” in which neoplastic cells infiltrate the choroid and/or sclera.

### Data analysis

**Figure 2.**
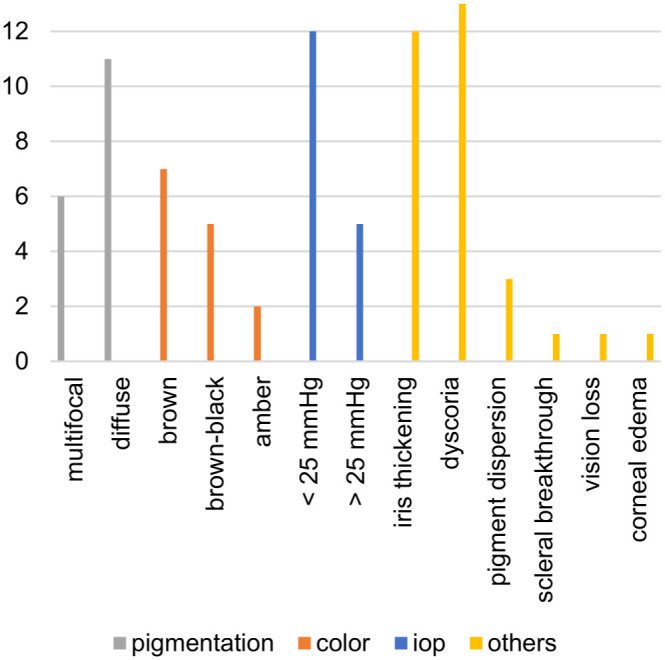
Clinical findings in 17 feline globes with iridal changes presumed to be feline diffuse iris melanoma by the contributing clinician. Y-axis shows the number of globes affected. IOP, intraocular pressure.

**Figure 3.**
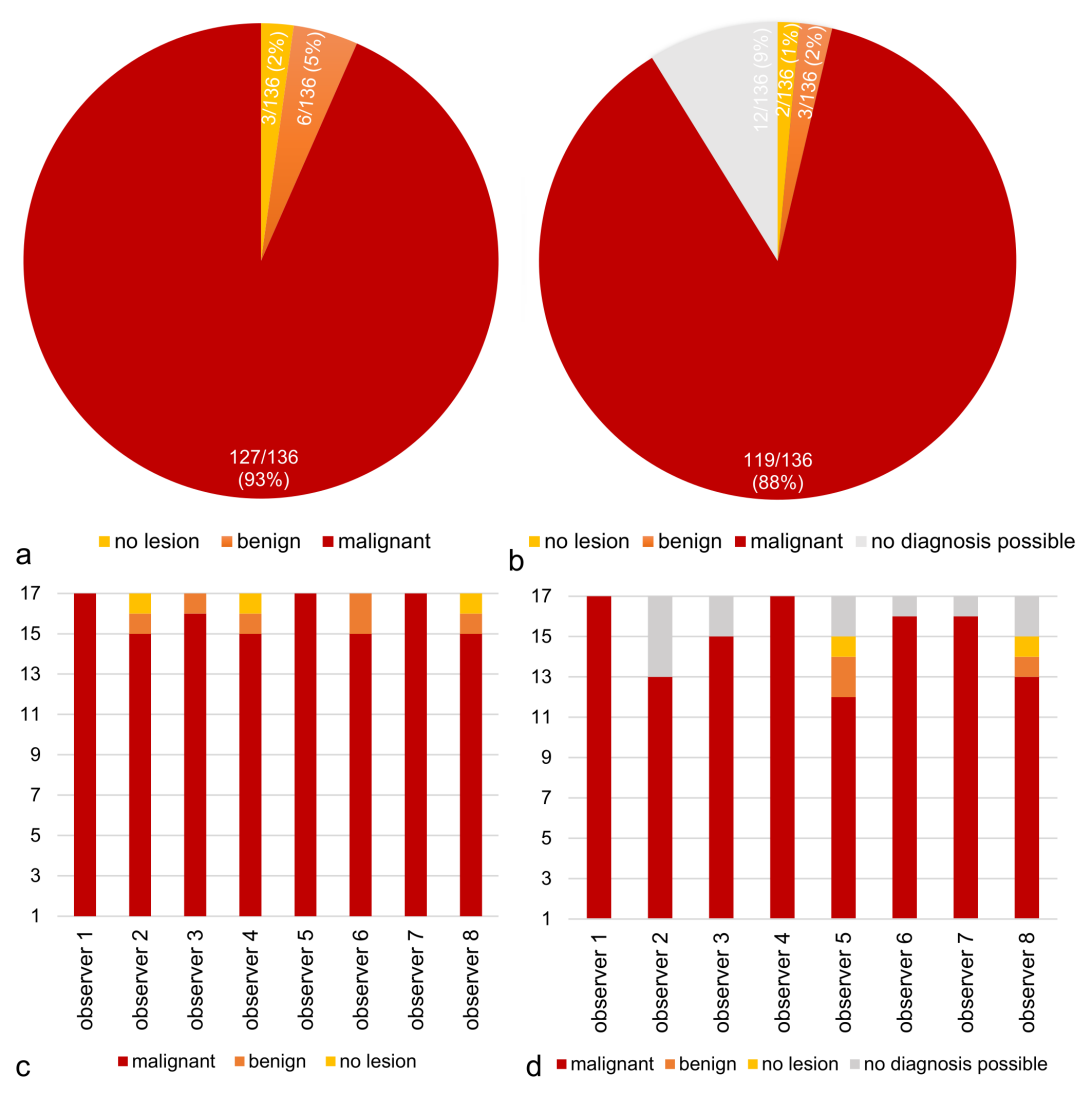
(a) Classification of histopathological diagnoses of the 17 feline globes made by 8 examiners (*n* = 136 assessments). (b) Classification of histopathological diagnoses of the iris biopsies made by 8 examiners (biopsies from 17 globes; *n* = 136 assessments). (c) Classification of histopathological diagnoses of the 17 feline globes parsed by observer. (d) Classification of histopathological diagnoses of the iris biopsies parsed by observer. Only samples for which a distinct diagnosis was possible (ie, not considered “not analyzable”) are shown.

**Figure 4.**
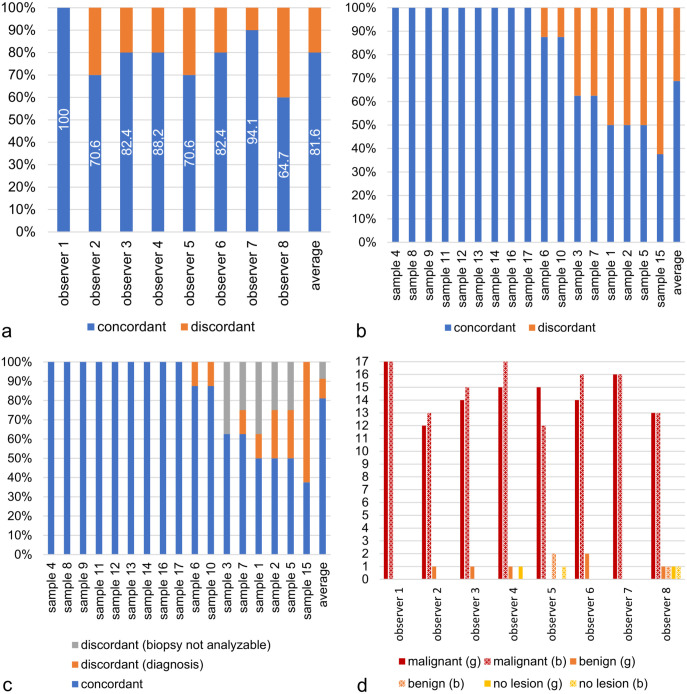
(a) Intraobserver concordance of the histopathological diagnosis for all 17 sample pairs (whole globe and corresponding iris biopsies) for each of the 8 examiners. Biopsies with no diagnosis due to technical artifacts and/or insufficient sample material were included. (b) Intraobserver concordance of the histopathological diagnosis for each of the 17 sample pairs (whole globe and corresponding iris biopsies). Biopsies with no diagnosis due to technical artifacts and/or insufficient sample material included. (c) Intraobserver concordance of the histopathological diagnosis for each of the 17 sample pairs (whole globe and corresponding iris biopsies). Biopsies with no diagnosis due to technical artifacts and/or insufficient sample material excluded. (d) Column chart of the histologic diagnoses given for the globe (g, *n* = 124) and the corresponding biopsy (b, *n* = 124) for each examiner. Pairings in which the examiner did not select a diagnosis due to technical artifacts and/or insufficient sample material for the biopsy are excluded (*n* = 12 from the total of 136).

Intraobserver agreement or disagreement was assessed exclusively, while interobserver agreement was not evaluated. A gold standard diagnosis was not factored in, and the initial diagnosis made by the routine diagnostic service was not cross-referenced with the diagnoses in this study. Concordance was defined as the complete agreement between the diagnoses of the globe and the corresponding biopsy. The data were analyzed descriptively, focusing on averages, frequencies, and percentages. The decision to perform a descriptive analysis was based on the fact that statistical methods assess how much of the variability in the data can be attributed to the observers; however, since there was little variability in our data (the diagnoses were largely consistent and predominantly positive [melanoma]), a meaningful statistical decomposition was not possible. A total of 272 diagnoses were included, comprising 136 diagnoses on each globe and iris biopsy slide. Descriptive analyses and graphical visualizations (Figs. 2–4) were performed using Microsoft Excel (Office 2019).

## Results

A total of 17 feline globes and their corresponding iris biopsies met the inclusion criteria and were included in the study. The represented breeds included domestic short hair (*n* = 12), Maine coon (*n* = 2), British short hair (*n* = 1), Devon rex (*n* = 1), and Siamese mix (*n* = 1). The median age was 8 years (range = 4–16 years) with 10 castrated males and 7 spayed females.

### Results of the Clinical and Ophthalmological Examinations

The lesions were diffuse (*n* = 11) or multifocal (*n* = 6). Hyperpigmentation was unilateral (*n* = 15) or bilateral (*n* = 2). The color was brown (*n* = 7), brown-black (*n* = 5), or amber (*n* = 2). In 3 cases, no information regarding lesion color was provided by the submitting ophthalmologists. In 12 cases, there was iris thickening, and in 13 cases, there was reduced pupillary mobility and/or dyscoria. Information on the iridocorneal angle was available in 13 of 17 cases. Seven of these 13 cases displayed pathologic changes (eg, pigment deposit, mass intruding the angle), and 6/13 were normal. Secondary glaucoma was found in 5/17 cases based on elevated IOP. The IOP of the affected eye ranged from 10 to 80 mm Hg. Further findings of the ophthalmological examination revealed vision loss (*n* = 1), scleral breakthrough (*n* = 1), generalized corneal edema in a patient with an IOP of 73 mm Hg (*n* = 1), and/or pigment dispersion (*n* = 3) within the aqueous humor (Fig. 2).

A chest radiograph was performed in 13/17 patients, and an abdominal ultrasound was performed in 11/17 patients. In 1 cat, a soft tissue-dense shadow was detected in the caudodorsal lung field on radiography, prompting further evaluation with computed tomography. The computed tomography scan revealed a contrast-enhancing lesion measuring approximately 1.5 × 2 × 2 cm; however, the owners declined a biopsy of the affected lung.

### Results of Histological Examinations

Detailed data on the histopathological findings, both in the globes and within the biopsies recognized by the 8 examiners, are presented in Table 1.

**Table 1.** Number of reports and percentage of histologic findings for biopsies (*n* = 17) and globes (*n* = 17) across the individual histopathological parameters of all 8 observers.

		Globe (*n* = 136)	Biopsy (*n* = 136)
Parameter	Category	(17 Slides × 8 Examiners)	
Analyzability (MC)	Yes	n/a	67 (49%)
	Yes, but with technical artifacts	n/a	52 (38%)
	No, not enough tissue	n/a	11 (8%)
	No, too many artifacts	n/a	17 (13%)
Invasion of neoplastic cells (MC)	Limited to the anterior surface	8 (6%)	10 (7%)
	Progression into the iris stroma (>3 layers)	65 (48%)	82 (60%)
	Complete obliteration of the iris stroma	75 (55%)	48 (35%)
	Obstruction of anterior chamber angle	48 (35%)	n/a
	Infiltration into the sclera	39 (29%)	n/a
	Invasion into the choroid	55 (41%)	n/a
	Others	31 (23%)	20 (15%)
	Not applicable	4 (3%)	13 (10%)
Arrangement of neoplastic cells (MC)	Single cells	11 (8%)	14 (10%)
	Clusters	46 (34%)	61 (45%)
	Diffuse	80 (59%)	56 (41%)
	Others	5 (4%)	15 (11%)
	Not applicable	3 (2%)	12 (9%)
Pigmentation	Mild	36 (26%)	20 (15%)
	Strong	82 (60%)	90 (66%)
	Not applicable	3 (2%)	12 (9%)
Cellular pleomorphism	Mild	77 (57%)	73 (54%)
	Strong	39 (29%)	30 (22%)
	Others	18 (13%)	18 (13%)
	Not applicable	3 (2%)	16 (12%)
Nuclear shape	Mild anisokaryosis	87 (64%)	74 (54%)
	Severe anisokaryosis	35 (26%)	28 (21%)
	Atypical nucleoli	35 (26%)	27 (20%)
	Others	31 (23%)	30 (22%)
	Not applicable	3 (2%)	17 (13%)
Final diagnosis (SC)	No lesion	3 (2%)	2 (1%)
	Melanosis	6 (4%)	3 (2%)
	Melanoma	127 (94%)	119 (88%)
	No diagnosis possible	0 (0%)	12 (9%)

In most categories, multiple answers (MC) were possible; SC, single answer; n/a, not applicable.

For the biopsy slides, “not analyzable” was selected 11/136 times due to not enough tissue. This involved 5/17 samples and was selected by 5/8 examiners. A maximum of 3/8 examiners made this selection for each sample that was difficult to analyze (eg, sample 1: 3/8, sample 2: 2/8, sample 7: 3/8). In 17 slides, the biopsy samples were marked as “not analyzable” due to too many artifacts. This concerned 6 samples and was declared by 8/8 examiners. A maximum of 6/8 examiners made this selection per sample (eg, sample 1: 6/8, sample 2: 5/8, sample 3: 4/8). Nevertheless, in 7 biopsy slides (2 = “no, too many artifacts” and 5 = “no, not enough tissue”), a diagnosis was still provided in the final section of the evaluation form. Four of 8 examiners provided a suspected diagnosis, even though they had deemed the sample unanalyzable.

Since the differentiation between early, mid-stage, and advanced FDIM is not possible based on biopsy samples, all subcategories of FDIM for the globes and the biopsies were grouped under the general term “malignant” to allow for consistent comparison between biopsy and globe diagnoses. As a result, 3 diagnostic categories were used for both globes and biopsies: no lesion, benign, and malignant. Regarding the biopsies, a fourth category was included to account for slides, in which no diagnosis could be made due to artifacts or insufficient tissue.

Of the 3 possible diagnoses for the entire globe, the following histopathologic diagnoses were given by the examiners (8 examiners × 17 slides = 136): no lesions in 3/136 slides (2.2%), benign melanosis in 6/136 slides (4.4%), and malignant alterations in 127/136 slides (93.4%) (Fig. 3a).

Of the 4 possible diagnoses for the biopsies, the following histopathologic diagnoses were chosen by the examiners: no lesion in 2/136 slides (1.5%), benign melanosis in 3/136 (2.2%), and malignant changes in 119/136 (87.5%). In 12/136 biopsy slides (8.8%), no diagnosis was selected due to technical artifacts and/or insufficient sample material (Fig. 3b).

The histological evaluation of the globes revealed that 3 observers diagnosed all 17 samples as malignant. “No lesion” was selected as a diagnosis in only 3 instances, while benign changes were identified in a total of 6 (Fig. 3c). In contrast, for the biopsies, 2 observers diagnosed all 17 samples as malignant. However, in 12 assessments, no diagnosis was possible due to technical issues or insufficient tissue material. In addition, “no lesion” was reported in 2 instances, and benign changes were identified in 3 assessments (Fig. 3d).

When comparing the histopathological diagnoses between the globe and the corresponding biopsy for each examiner, identical diagnoses were made in 11 to 17 of the 17 cases (average 13.8) (biopsies with the diagnosis “no distinct diagnosis due to technical artifacts and/or insufficient sample material” included). Detailed information on the average and range of identical diagnoses across the various histopathological parameters for the identical biopsy (*n* = 17) and globe (*n* = 17) of all 8 observers in provided in Table 2.

**Table 2.** Average values and ranges for identical histopathological parameters and final diagnoses recorded for the 17 globes and corresponding 17 iris biopsies, as evaluated by 8 observers.

		Average	Range
Parameter	Category	(of the Identical Diagnoses for the Globe and Corresponding Biopsy) Total Number of Cases: 17	
Invasion	Limited to the anterior surface	0.13	0-1
	Progression into the iris stroma (>3 layers)	5.50	1-16
	Complete obliteration of the iris stroma	5.25	3-10
Arrangement of neoplastic cells	Single cells	0.25	0-1
	Clusters	4.00	0-9
	Diffuse	6.60	3-17
Pigmentation	Mild	1.25	0-3
	Strong	7.30	3-14
	Others (moderate)	0.75	0-4
Cellular pleomorphism	Mild	7.25	0-14
	Strong	3.25	1-13
	Others (moderate)	0.75	0-3
	Others (few anisocytic cells)	0.13	0-1
Nuclear shape	Mild anisokaryosis	7.50	0-13
	Severe anisokaryosis	2.75	1-13
	Atypical nucleoli	2.75	0-14
	Others (moderate)	0.63	0-3
	Others (few anisokaryotic cells)	0.13	0-1
	Others (multinucleated cells)	0.25	0-1
	Others (karyomegaly)	0.13	0-1
Final diagnosis		13.8	11-17

The reported values represent the mean number of identical assessments per case across all observers. Biopsies with no diagnosis due to technical artifacts and/or insufficient sample material were included in the analysis.

### Concordance

The intraobserver concordance between final diagnoses for the globes and corresponding iris biopsies ranged from 64.7% to 100%, with an average concordance of 81.6% (Fig. 4a). For globes with early changes, the concordance ranged from 50% to 100% (average 79.7%). For globes with advanced stages of FDIM, the concordance ranged from 37.5% to 100% (average 91.1%).

The observer with a concordance of 100% consistently selected “malignant” in all cases (for both globes and biopsies). The highest discordance was observed with observers 2, 5, and 8. Observer 2 classified the corresponding biopsy as “not analyzable” in 4 out of the 5 assessments in which their diagnoses did not match. For observer 5, this occurred in 2 out of 5 instances, and for observer 8, in 2 out of 6 instances.

Examining the intraobserver concordance from the perspective of the individual sample pairs, the concordance ranged from 37.5% to 100%, with an average of 68.8%. In 9/17 sample pairings (52.9%), the concordance of the diagnosis for the globe and the corresponding biopsy was 100%. In 8/17 sample pairings (47.1%), the concordance ranged from 37.5 to 87.5%. Sample 15 showed the lowest concordance, with only 3 examiners making the same histopathological diagnosis for the globe and the biopsy; the remaining 5/8 observers classified the globe as benign and the corresponding biopsy as malignant. Discordance occurred in 5/17 samples due to biopsies that could not be analyzed (Fig. 4b, c).

Figure 4d provides a direct comparison of the results from the perspective of each observer; however, pairings in which the biopsy was declared “not analyzable” were excluded from this analysis. This adjustment reveals a 100% concordance between the diagnoses of observer 1 and observer 7.

## Discussion

This study represents the first direct comparison of iris biopsies and enucleated globes in assessing FDIM. It fills a critical gap in the published veterinary literature, particularly by evaluating the diagnostic reliability of biopsies as a less invasive alternative to immediate enucleation. Eight board-certified pathologists, including ocular pathologists, contributed to the study, ensuring diverse expertise. No notable differences in intraobserver concordance were observed between ocular and general pathologists, and for this reason, subgroup analysis was not further pursued. A key finding was the strong influence of tissue conditions following biopsy on analyzability, which is likely associated with the biopsy retrieval technique. In total, 12 out of 136 biopsy evaluations were deemed non-diagnostic due to technical artifacts or insufficient sample material. The general concordance rate of histopathological diagnoses was 81.6%, with intraobserver agreement varying between 64.7% and 100%.

Clinical assessment of FDIM is inherently challenging. The PPSS proposed by Fuchs et al^9^ offers a valuable advancement. This system provides a structured framework to grade the severity of iris pigmentation based on clinical parameters such as pigmentation extent, pupil mobility, and IOP. By incorporating these features, PPSS enables a more objective stratification of FDIM severity compared to historical reliance on subjective visual assessments. Unfortunately, the paper written by Fuchs et al was published after the conduction of our study. Therefore, the PPSS was not used when clinically assessing the cases included. A limitation of the PPSS is its exclusion of gonioscopic findings, which could greatly enhance diagnostic precision. Gonioscopy provides direct visualization of the iridocorneal angle, identifying critical changes like pigment deposition, tumor invasion, and/or angle obstruction—key features for distinguishing benign melanosis from progressive FDIM. It is highly recommended to use the PPSS including gonioscopic findings in future studies concerning FDIM.

This study partially supports that iris biopsies can complement clinical assessment when the retrieved tissue is of sufficient quality, with early-stage FDIM being diagnosed with a 79.7% concordance and advanced-stage FDIM with 91.1% concordance.

The focus of the presented study on cases with suspected malignancy fits its diagnostic goals but limits the evaluation of biopsy reliability for benign or healthy tissue. Including 3 balanced groups (healthy, benign, and malignant) would offer a more comprehensive understanding of biopsy performance across different clinical scenarios. However, the intentional inclusion of healthy tissue was never a consideration in this study, as the goal was not to assess whether pathologists can identify normal tissue. Such an approach would not reflect the realistic diagnostic challenges of distinguishing between benign and malignant changes. The low number of melanosis cases (only 2) is due to the selection of samples from cats that were enucleated because of suspected FDIM, resulting in very few negative (melanosis) samples. Notably, only one of the 8 observers agreed with the diagnosis of melanosis in the biopsy. In comparable human medical studies, healthy tissue is also not included, as these studies typically focus on cases with suspected malignancy.^8,18^ This parallels the approach in this study, emphasizing the clinical need to address ambiguous or potentially malignant cases rather than normal tissue. These observations underline the appropriateness of the sample selection while recognizing the limitations in evaluating benign tissue.

The histological evaluations in this study revealed several challenges, particularly in smaller or artifact-prone biopsy samples. Observers noted discrepancies in diagnoses, often attributable to insufficient sample quality or lack of representativeness. As expected, cases with complete diagnostic concordance were typically associated with more advanced disease stages, where histopathology criteria of malignancy are easier to detect.

The reliance on single-section analysis per globe, while practical, represents a significant limitation. Subtle or localized changes may have been missed, reducing the detection rate of minimal or early-stage alterations. Some pathologists contributing to this study indicated a preference for additional slides from multiple blocks to allow for more detailed evaluations. Clearly, serial sectioning enhances the detection of subtle pathological changes. When planning a study, the balance between sample size, workload, and possible significance of the results needs to be considered. In our study, the aims to include a fair number of participating pathologists and a broad range of cases were prioritized above the benefit of multiple slides per case.

The samples in this study were available exclusively as whole-slide images. Reviewing the original glass slides might have been advantageous, as some sections appeared out of focus to 1 observer. However, no other observers raised this concern for any sample, and to ensure consistency, no additional slides were provided. Current literature supports the diagnostic equivalence of digital slides and traditional glass slides.^1,11^ Digital pathology offers advantages such as enhanced image accessibility and analysis efficiency, although minor limitations like focus variability can occasionally arise.^1^

Generally speaking, the diagnostic yield of biopsies is heavily influenced by factors such as size, quality, and tissue handling. In ophthalmology, the orbital anatomy and globe mobility limit the accessibility of iris biopsy sites.^8^ The presented study benefited from controlled conditions using enucleated eyes, allowing for optimal biopsy retrieval. As a result, the size of the samples obtained contrasts with typical clinical submissions seen by the ocular pathologists contributing to our study (ES and CN), with the samples in this study being larger. However, for 11 out of 136 histological examinations of the biopsy slides, no definitive diagnosis could be established. This highlights the crucial need for clinicians to be aware of the impact of iris biopsy retrieval and tissue handling on the diagnostic power of the biopsy.

The clinical relevance of this study lies in the assessment of the diagnostic reliability of iris biopsies, providing clinicians with valuable information to determine whether a biopsy is an appropriate diagnostic choice for their patient with suspected FDIM. With an average concordance rate of 81.6% and 12 out of 136 examinations of the biopsy slides being deemed non-diagnostic due to technical artifacts or insufficient tissue, the results highlight the challenges inherent in relying on biopsies for definitive diagnoses. Notably, the majority of the globes examined in this study exhibited mid to advanced stages of FDIM, where histopathological features are more pronounced and more easily identifiable. However, clinicians must weigh the benefits and limitations of iris biopsies in their decision-making process. The study’s findings suggest that the diagnostic value of iris biopsies may not be universally sufficient, particularly in cases with poor sample quality. Therefore, each clinician must carefully evaluate whether the accuracy provided by iris biopsies, given these potential limitations, is adequate for the specific clinical scenario.

A potential direction for future studies would involve a prospective design with a larger sample size, incorporating a wider range of disease stages, particularly focusing on melanosis and early-stage FDIM cases. In clinical practice, biopsies are primarily submitted when there is uncertainty whether a lesion is benign melanosis or an early-stage FDIM. Once the disease progresses to more advanced stages, where clinical signs such as iris thickening, dyscoria, and/or secondary glaucoma are present, biopsies are less likely to be performed, as the diagnosis is more straightforward based on clinical findings and imaging. Therefore, future studies should ideally focus on cases of melanosis and early FDIM, as this would allow for a more targeted assessment of the concordance between biopsy and enucleated globe in these specific stages of the disease. The current study, while valuable, did not provide sufficient representation of these cases due to the inclusion of predominantly moderate to advanced FDIM cases, which limits the applicability of the findings to early-stage lesions. Focusing on these earlier stages in future research could provide critical insights into the diagnostic accuracy of iris biopsies in distinguishing between benign and malignant changes, thus enhancing the clinical utility of this technique.

The clinical implementation of iris biopsies in veterinary practice remains a procedure likely to be reserved for experienced ophthalmologists due to its technical complexity and potential risks. The procedure requires a high level of skill to ensure adequate tissue handling. Risks associated with iris biopsies include intraocular hemorrhage, damage to ocular structures such as the lens or retina, infection, and the potential for increased IOP leading to secondary glaucoma.^8^ In addition, the manipulation of the iris may lead to uveitis or even lens injury in some cases.^17^ These risks highlight the importance of performing the procedure under controlled conditions by a skilled ophthalmologist, ideally in a referral or specialized setting, to minimize complications and ensure optimal diagnostic yield. A recent study on diode laser ablation for treating progressive pigmented iris lesions in cats (317 cats, 356 eyes) by Fuchs et al^9^ showed it to be a safe and effective alternative to iris biopsy and/or enucleation. While laser therapy can reduce pigment progression and maintain ocular function, it does not provide histopathological insight into whether the lesion is benign melanosis or malignant FDIM, which is a limitation compared to biopsies. This makes the choice between laser therapy and biopsy a matter of clinical preference, as both options are practical in early-stage disease. For advanced cases, however, laser therapy may require repeat treatments, and enucleation may be necessary. Both procedures have their merits, but iris biopsies provide more definitive diagnostic information. The decision largely depends on the clinician’s assessment of the lesion and the patient’s overall condition.

A limitation of this study is the relatively small sample size of 17 globes and corresponding biopsies. As a result, only descriptive analyses were performed, and no inferential statistical comparisons were made. The limited number of cases restricts the generalizability of the findings; however, the study provides valuable insights into the histological concordance between iris biopsies and enucleated globes. These findings may serve as a basis for future studies with larger case numbers.

Another limitation of this study is that diagnostic accuracy metrics such as sensitivity and specificity could not be calculated, as there was no independent gold standard for comparison. While intraobserver concordance between the biopsy and globe diagnoses provides insight into internal consistency, it does not allow definitive conclusions about diagnostic correctness. Therefore, concordance should not be equated with accuracy.

## Conclusion

Iris biopsies offer a valuable diagnostic tool for identifying FDIM. The present study showed an average histological concordance of 81.6% between biopsies and enucleated globes, supporting their utility in more advanced stages where changes are more evident. However, the reliability of biopsies is impacted by factors such as tissue quality and biopsy technique. These limitations emphasize the need for skilled execution and suggest that iris biopsies should be performed by experienced ophthalmologists. Future research should focus on including a broader range of disease stages, especially early FDIM and melanosis, to better assess the performance of biopsies across different clinical scenarios. Given the relatively small sample size, the findings of this study should be regarded as exploratory. Despite these challenges, iris biopsies offer an important diagnostic alternative to immediate enucleation, with the potential to guide clinical decisions in cases where early intervention is necessary.

## References

[1] Al-Janabi S Huisman A Vink A , et al. Whole slide images for primary diagnostics in dermatopathology: a feasibility study. J Clin Pathol. 2012;65:152–158. 22031590 10.1136/jclinpath-2011-200277

[2] Ben-Shlomo G. Iris Freckles, Nevi, & Melanosis. ISU. January, 2019. Accessed February 21, 2023. https://www.cliniciansbrief.com/article/iris-freckles-nevi-melanosis.

[3] Day MJ Lucke VM. Melanocytic neoplasia in the cat. J Small Anim Pract. 1995;36:207–213. 7650915 10.1111/j.1748-5827.1995.tb02898.x

[4] Dubielzig RR . Tumors of the eye. In: Meuten DJ , ed. Tumors in Domestic Animals. 5th ed. Hoboken, NJ: John Wiley & Sons Inc; 2017: 892–922.

[5] Dubielzig RR Ketring KL McLellan GJ , et al., eds. Veterinary Ocular Pathology: A Comparative Review. Amsterdam, The Netherlands: Elsevier; 2010.

[6] Duncan DE Peiffer RL. Morphology and prognostic indicators of anterior uveal melanomas in cats. Prog Vet Comp Ophthalmol. 1991;1:25–32.

[7] Featherstone HJ Scurrell EJ Rhodes M , et al. Iris biopsy to investigate feline iris hyperpigmentation. Vet Ophthalmol. 2020;23:269–276. 31733046 10.1111/vop.12718

[8] Finger PT Milman T. Microincision, aspiration cutter-assisted multifocal iris biopsy for melanoma. Eur J Ophthalmol. 2017;27:62–66. 27228972 10.5301/ejo.5000809

[9] Fuchs AA Giuliano EA English R , et al. Diode laser ablation of progressive pigmented iris lesions in 317 cats (356 eyes) appears overall safe and effective in decreasing progression of iris pigmentation. J Am Vet Med Assoc. 2024;262:117–124. 37758183 10.2460/javma.23.07.0387

[10] Glaze MB Maggs DJ Plummer CE . Feline ophthalmology. In: Gelatt KN , ed. Veterinary Ophthalmology. 6th ed. Hoboken, NJ: Wiley; 2021: 1665–1840.

[11] Hanna MG Monaco SE Cuda J , et al. Comparison of glass slides and various digital-slide modalities for cytopathology screening and interpretation. Cancer Cytopathol. 2017;125(9):701–709. 28558124 10.1002/cncy.21880

[12] Kalishman JB Chappell R Flood LA , et al. A matched observational study of survival in cats with enucleation due to diffuse iris melanoma. Vet Ophthalmol. 1998;1(1):25–29. 11397206 10.1046/j.1463-5224.1998.00006.x

[13] Kayes D Blacklock B. Feline uveal melanoma review: our current understanding and recent research advances. Vet Sci. 2022;9(2):46. 35202299 10.3390/vetsci9020046 PMC8877522

[14] Linn-Pearl RN Powell RM Newman HA , et al. Validity of aqueocentesis as a component of anterior uveitis investigation in dogs and cats. Vet Ophthalmol. 2015;18(4):326–334. 25557502 10.1111/vop.12245 PMC7169297

[15] Mould JR Peterson-Jones SM Peruccio C , et al. Uveal melanocytic tumors. In: Peiffer RL Simons KB , eds. Ocular Tumors in Animals and Humans. 1st ed. Ames: Iowa State Press; 2002: 225–281.

[16] Olin DD. Examination of the aqueous humor as a diagnostic aid in anterior uveitis. J Am Vet Med Assoc. 1977;171(6):557–559. 578803

[17] Patnaik G Annamalai R Biswas J. Intraocular biopsy in uveitis. Indian J Ophthalmol. 2020;68(9):1838–1843. 32823400 10.4103/ijo.IJO_1325_20 PMC7690489

[18] Priemer DS Curran JM Phillips CL , et al. Concordance of solid organ biopsy diagnoses with hospital autopsy and the contribution of biopsies to death. Cureus. 2023;15(1):e33889. 10.7759/cureus.33889 PMC9934933 36819431

[19] Smith SH Goldschmidt MH McManus PM . A comparative review of melanocytic neoplasms. Vet Pathol. 2002(39):651–678. 10.1354/vp.39-6-651 12450197

[20] Wiggans KT Reilly CM Kass PH , et al. Histologic and immunohistochemical predictors of clinical behavior for feline diffuse iris melanoma. Vet Ophthalmol. 2016;19(Suppl 1):44–55. 26805705 10.1111/vop.12344

[21] Wiggans KT Vernau W Lappin MR , et al. Diagnostic utility of aqueocentesis and aqueous humor analysis in dogs and cats with anterior uveitis. Vet Ophthalmol. 2014;17(3):212–220. 23910096 10.1111/vop.12075 PMC7169337

